# Manipulating Topological States by Imprinting Non-Collinear Spin Textures

**DOI:** 10.1038/srep08787

**Published:** 2015-03-05

**Authors:** Robert Streubel, Luyang Han, Mi-Young Im, Florian Kronast, Ulrich K. Rößler, Florin Radu, Radu Abrudan, Gungun Lin, Oliver G. Schmidt, Peter Fischer, Denys Makarov

**Affiliations:** 1 Institute for Integrative Nanosciences, IFW Dresden, 01069 Dresden, Germany; 2 Center for X-ray Optics, Lawrence Berkeley National Laboratory, Berkeley CA 94720, USA; 3 Daegu Gyeongbuk Institute of Science and Technology, Daegu, Korea; 4 Helmholtz-Zentrum Berlin für Materialien und Energie GmbH, 12489 Berlin, Germany; 5 Institute for Theoretical Solid State Physics, IFW Dresden, 01069 Dresden, Germany; 6 Institut für Experimentalphysik/Festkörperphysik, Ruhr-Universität Bochum, 44780 Bochum, Germany; 7 Material Systems for Nanoelectronics, Chemnitz University of Technology, 09107 Chemnitz, Germany; 8 Physics Department, UC Santa Cruz, Santa Cruz CA 95064, USA

## Abstract

Topological magnetic states, such as chiral skyrmions, are of great scientific interest and show huge potential for novel spintronics applications, provided their topological charges can be fully controlled. So far skyrmionic textures have been observed in noncentrosymmetric crystalline materials with low symmetry and at low temperatures. We propose theoretically and demonstrate experimentally the design of spin textures with topological charge densities that can be tailored at ambient temperatures. Tuning the interlayer coupling in vertically stacked nanopatterned magnetic heterostructures, such as a model system of a Co/Pd multilayer coupled to Permalloy, the in-plane non-collinear spin texture of one layer can be imprinted into the out-of-plane magnetised material. We observe distinct spin textures, *e.g.* vortices, magnetic swirls with tunable opening angle, donut states and skyrmion core configurations. We show that applying a small magnetic field, a reliable switching between topologically distinct textures can be achieved at remanence.

Magnetisation configurations with nontrivial topological properties manifest an enhanced stability under external perturbations^1^,^2^,^3^. Whereas vortices form in soft-magnetic confined films^4^ due to minimisation of exchange and stray field energy, more recently discovered topological states, such as *chiral skyrmions*^5^,^6^,^7^, are stabilised by the antisymmetric magnetic exchange, known as Dzyaloshinskii-Moriya interaction (DMI)^8^. The chiral skyrmions in magnetic systems with defined handedness provide countable units characterised by a topological charge. This integer index describes the fact that a skyrmion cannot be unwound by any continuous deformation of the magnetisation. Conduction electrons passing through a non-collinear spin texture with a topological charge density pick up a Berry phase, which can give rise to an anomalous *topological* Hall effect^9^,^10^,^11^,^12^,^13^. Skyrmion systems composed of solitonic units can be assembled into arrays which display an additive behaviour of the net Berry phase. Controlling skyrmionic states is becoming a focus of intense research both for fundamental understanding^1^,^2^,^6^,^7^,^14^ and potential applications^15^,^16^. So far, chiral skyrmions have been only observed in single-phase materials with acentric crystal structures^5^,^6^,^17^,^18^,^19^,^20^ or in magnetic films owing to broken inversion symmetry^7^,^14^,^21^,^22^ and at low temperatures that severely limits their potential application in spintronics.

An alternative route to exploit topological properties of magnetisation distributions is to create magnetic heterosystems exhibiting non-collinear spin textures. In particular, these nontrivial topological states generate a topological charge density which instills Berry phases and transport anomalies. Recent numerical simulation studies have suggested the possibility to build up skyrmion configurations in magnetic systems without DMI *via* interlayer exchange coupling in thin soft-magnetic bilayers^24^ or in out-of-plane magnetized films with patterns of soft-magnetic disks on top^23^. State-of-the-art nanofabrication allows for designing such functional heterostructures with tunable properties of the subsystems and an enhanced stability of the entire structure^25^,^26^. The advantage of this approach is to utilise conventional magnetic materials and to adjust key parameters, such as anisotropy or coupling constants in a wide range by tailoring the geometry of the heterostructures. The recent experimental report on artificial magnetic skyrmions at room temperature represents a first step towards their realisation^27^.

Here, we propose theoretically and demonstrate experimentally nontrivial topological states by imprinting non-collinear spin textures into an out-of-plane magnetised material *via* stacking two magnetic nanopatterns consisting of layers with in-plane and out-of-plane magnetisation. Within such heterostructures, it is possible to create vortex, spiral domain, and skyrmion states with different topological charge densities in the originally out-of-plane magnetised layer [Fig. 1]. In confined nanostructures, like circular dots, non-collinear magnetization configurations can be induced and shaped as a consequence of the dipolar stray field, which enforces magnetic flux closure, and exchange coupling between different parts of the heterostructure. A topologically stable state in strict sense cannot exist in a confined magnetised body with open boundaries, because a nontrivial magnetization configuration always can be destroyed by moving it across the boundary. This instable topology of any state in a confined body can formally be understood by considering the elementary classification of topological states through homotopy groups^28^. The maps from closed loops or surfaces in the physical space to the order-parameter space, which for the magnetisation vector is the sphere spanned by its directions, can be continuously moved across the boundaries of the body. Thus, an unambiguous definition of a stable topology is impossible [see Supplementary for further details]. However, topological charge densities and their integrals are well defined and are physical observables. They are not conserved topological indices with integer values, but the topological charges are geometric properties of the magnetisation configuration, measured by rational numbers. In this sense, the magnetic states in a confined nanoobject can be distinguished and their similarity with corresponding topologically nontrivial states in an infinite system can be quantified. Therefore, we address configurations in Co/Pd sub-structure with a net skyrmion charge *S* ≈ ±1 as skyrmion cores. Similarly, the vortices in such dots have 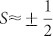 and deviate from the real vortex defect state of a planar spin system due to the presence of a tilt of magnetization at the edge.

We address the most crucial aspects, namely, how to control these states and to manipulate the respective topological charge densities. Spin textures can possess distinct topological charge densities, defined as the skyrmion number 
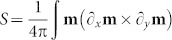
 d*x*d*y* with the magnetisation *m* = *M*/*M_S_* of the out-of-plane magnetised layer. In the skyrmion states, the net integral charge can be fractional owing to the confinement within the nanostructure. The net charge is governed by an opening angle of the spin texture that can be precisely controlled by tuning the interlayer exchange coupling between in-plane and out-of-plane magnetic substructures and applying small magnetic fields. Our concept is experimentally confirmed by observing the magnetic configurations and magnetisation reversal processes in heterostructures consisting of nanopatterned out-of-plane magnetised Co/Pd films and Permalloy (Py, Ni_80_Fe_20_) nanostructures in the vortex state separated by a thin Pd spacer. Two magnetic X-ray microscopy techniques, namely bulk sensitive magnetic transmission soft X-ray microsopy (MTXM) and surface sensitive X-ray photoemission electron microscopy (XPEEM), were used to image the magnetic domains in the individual layers, harnessing the inherent element-specificity of the X-ray magnetic circular dichroism (XMCD) effect. Furthermore, XMCD spectroscopy enabled us to retrieve element-specific hysteresis curves in those structures. We prove that the interlayer coupling strength can be tailored by adjusting the thickness *d* of the non-ferromagnetic Pd spacer layer between Permalloy and Co/Pd layers.

## Theoretical predictions

**Figure 1:**
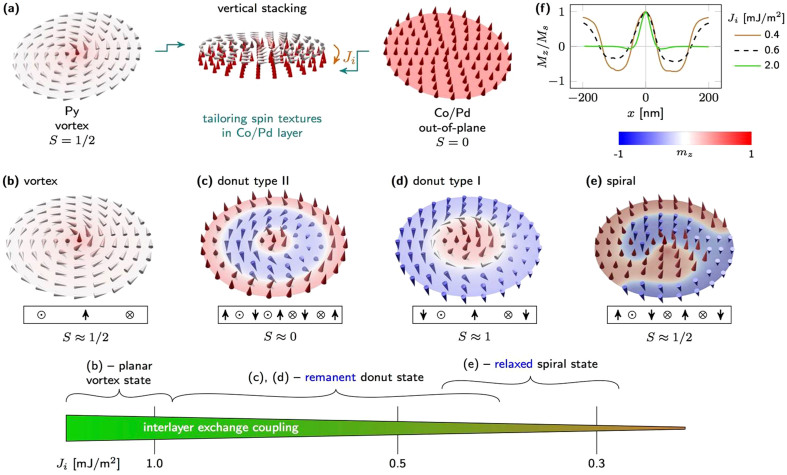
Imprinting non-collinear magnetic spin textures into out-of-plane magnetised films (Co/Pd multilayers) *via* interlayer coupling to a vortex state (Permalloy, Py) (a). Calculations are carried out for a Co/Pd anisotropy of *K_u_* = 200 kJ/m^3^. Depending on the interlayer exchange coupling strength and the magnetic field treatment (remanence or relaxed state), configurations of distinct topology can be imprinted in the out-of-plane magnetised layer as revealed by micromagnetic simulations. Figures (b)–(e) show the magnetic configuration of four different states in Co/Pd films with decreasing strength of interlayer coupling *J_i_* (left to right) after applying an out-of-plane magnetic field. Colours correspond to the normalised out-of-plane magnetisation component: (b) Remanent and relaxed vortex state; (c)–(d) remanent donut state type II and type I (number of domain walls), respectively; and (e) relaxed magnetic spiral. The skyrmion number *S* of each state is assessed based on the sketched magnetisation configuration in the cross-section. (f) Line profiles through the center of the Co/Pd film for the normalised out-of-plane magnetisation (*m_z_* = *M_z_*/*M_s_*) in remanent state Co/Pd spins illustrate the possibility to tailor the opening angle of the donut state by adjusting *J_i_*. The magnetic spiral also appears after opening the circular domain wall of the donut state by applying a small in-plane magnetic field.

Firstly, we investigate this concept by a *numerical study* of magnetic spin textures in a disk-like heterostructure consisting of a hard-magnetic out-of-plane magnetised Co/Pd multilayer stack and a soft-magnetic Permalloy that are separated by a non-magnetic spacer of varying thickness *d* [Fig. 1(a)]. The interlayer exchange coupling strength is varied in the range from *J_i_* = 0.1 to 2 mJ/m^2^ (Pd spacer thickness 0 to 30 nm), as expected *e.g.* for RKKY-type coupling through Pd^29^,^30^. The magnetic coupling through thick Pd spacers (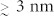) is mediated by spin diffusion mechanisms involving unfilled 4*d* and 5*s* − *p* Pd bands^29^, which can numerically described in the same way. Magnetostatic coupling alone does not stabilise the reported configurations [Supplementary Fig. 1]. We focus on the magnetic pattern in the Co/Pd multilayers, since the large asymmetry between Co/Pd and Py thickness preserves the topology of the Py vortex.

In strongly coupled systems (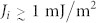), the equilibrium spin configuration, *i.e.* vortex structure, exhibits the same circulation (sense of rotation of in-plane magnetisation with respect to surface normal) in the Py and the Co/Pd layers. The minimum of the normal magnetisation *m_z_* around the core is enlarged compared to that of decoupled Py vortices.

Decreasing the interlayer exchange coupling and running major or minor hysteresis loops stabilise the remanent donut state type II [Fig. 1(c)] and type I [Fig. 1(d)] with two and one domain walls, respectively, and radially varying opening angle/out-of-plane magnetisation component [Fig. 1(f), also Supplementary Fig. 2]. In particular, donut type I exhibits a spin configuration similar to those in a disk with DMI^31^. In the view of the definition above, this state is referred to as a skyrmion core configuration. The sense of in-plane circulation remains as in the Py disk. The circular symmetry is caused by the imprint of the central vortex core region. The vortex core in the Co/Pd layer is more extended in weakly coupled systems (*J_i_* = 0.4 mJ/m^2^), since the ratio between interlayer coupling and out-of-plane anisotropy constant *K_u_* becomes smaller [Supplementary Fig. 2](j)]. For *J_i_* = 0.4 mJ/m^2^, the vortex core diameter in donut configurations is about 80 nm compared to 6 nm in Py, illustrating the possibility to tailor the vortex core profile *via* imprint, which might be beneficial for studying the inner structure of the vortex core. Systems with even smaller interlayer coupling (
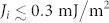
) exhibit a non-uniform single-domain remanent state [Supplementary Fig. 2(a)]. The remanent donut state type II cannot be obtained in systems with 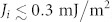, because all Co/Pd spins except the vortex core flip simultaneously.

Another distinct magnetic state is the spiral state [Fig. 1(e)] that can be set after relaxation from the out-of-plane saturated state [Supplementary Fig. 3]. Contrary to the remanent donut states, where the edge magnetisation is invariant under azimuthal rotation, the Co/Pd spins possess a sufficiently large energy during relaxation to modify the initial homogeneous magnetisation at the edge. For the formation of a spiral state, the starting configuration is a magnetic domain wall that is anchored at the edge in two points. The imprinted vortex core acts as a driving force that twists the domain wall to a spiral while approaching the center. As the ratio *J_i_*/*K_u_* increases, the spiraling distortion of the domain wall by the imprinted vortex is stronger leading to a more extended spiral. In this respect, its experimental observation would represent a strong hint for an interlayer exchange coupling with an in-plane circulation.

## Experimental observation

**Figure 2:**
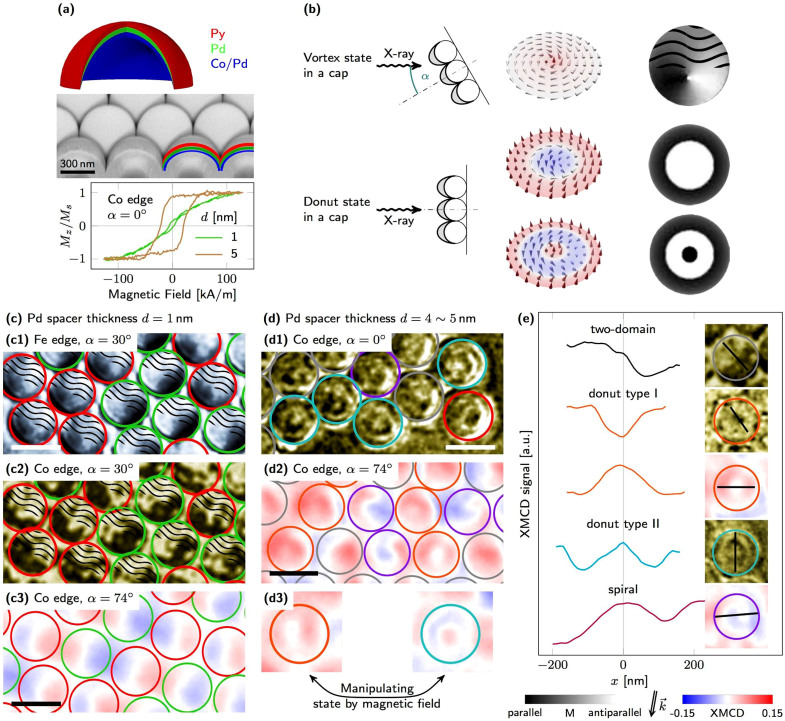
Visualising magnetic spin textures by means of MTXM and XPEEM. (a) Schematics of the investigated layer stack and SEM cross-section of the sample. Magnetic hysteresis loops of Co/Pd spins for different Pd spacer thickness *d* reveals peculiar properties of the approach. (b) Contrast origin for curved non-collinear textures (XMCD contrast calculated, not normalised). The wavy pattern indicates regions of very weak/absent XMCD contrast. (c), (d) MTXM (brown and gray shaded) and XPEEM (red-blue colorspace) images of the remanent state of closely packed CoPd/Pd(*d*)/Py caps with various Pd spacer thickness reveal non-collinear spin textures in Co/Pd. (c) For 1 nm Pd spacer thickness, Py and Co/Pd spins form a vortex with the same circulation (left and right circulation labelled by red and green circles). (d) Samples with 4 ~ 5 nm-thick Pd spacer show stronger out-of-plane contrast, and the numerically predicted states: red – donut state type I; cyan – donut state type II; purple – spiral state; gray – ambiguous. (See text for information about magnetic fields applied before.) The scale bar is 500 nm. (e) Line profiles. The core size of the donut state ranges from 60 to 110 nm. The XPEEM data within each cap exhibits a small asymmetry originating from a small in-plane magnetisation component of the Co/Pd spins. (In-plane contrast of images at Co Ledge is enhanced for better visualisation.)

These theoretical predictions are further *experimentally verified* by measuring the magnetisation reversal process and visualising the magnetisation texture in nanopatterned [Co/Pd]/Pd/Py multilayers. Tailoring the ratio *J_i_*/*K_u_* can experimentally be realised by modifying the thickness and material of the non-magnetic spacer or tuning the thickness of Co in the Co/Pd multilayers. Here, we modify the thickness of the non-magnetic spacer. Layer stacks consisting of Pd(2)/[Co(0.4)/Pd(0.7)]_5_/Pd(*d*)/Py(40)/Pd(2) with thicknesses in nm and variable Pd spacer thickness *d* ranging from 1 to 30 nm were prepared onto assemblies of non-magnetic SiO_2_ spherical particles with a diameter of 500 nm [Fig. 2(a)]. Reference samples with single Py and Co/Pd films are shown in Supplementary Fig. 4. Changing the spacer thickness at small values affects the magnetic properties of the Co/Pd spins significantly, transforming the originally out-of-plane preferential orientation into an in-plane preference [Supplementary Fig. 5]. Using magnetic caps instead of planar disks provides several experimental advantages. We already demonstrated that arrays of closely packed vortices can be achieved in Py cap structures^32^,^33^ in contrast to planar disks. Closely packed Py vortices with small intercap interaction provide a large signal when measuring the magnetisation reversal process and yield a better statistics during magnetisation visualisation in full-field magnetic microscopes. The curvature gradient of the cap reduces significantly the magnetostatic interaction with nearest neighbor caps^32^. The residual interaction is small and of dynamic nature: the circulations are predetermined during the vortex nucleation process (interaction between total magnetic moments of the neighbouring caps). Upon nucleation, the interaction between neighbouring caps is substantially reduced (interaction between vortex cores of the neighbouring caps). More importantly, patterning *via* electron beam lithography and lift-off or etching can lead to edge roughnesses that act as pinning sites, thus disturbing the formation of the desired non-collinear spin textures. Contrary, deposition of magnetic thin films onto arrays of spherical particles without any post-processing ensures highest possible structure quality. Furthermore, the angular spread of the out-of-plane easy axis of the Co/Pd spins on a cap^34^,^35^ provides a perspective to reduce the switching fields at the edge and thus the required field values to switch between donut type I and type II.

Utilising the element specificity of MTXM^36^ and XPEEM^37^, we unambiguously proved by two independent visualisation techniques and samples the stabilisation of non-collinear spin textures in Co/Pd layers. The magnetic contrast is provided by XMCD that detects the difference in X-ray absorption depending on the relative orientation between photon beam and sample surface^36^. In the MTXM, both out-of-plane and in-plane components of the magnetisation can be distinctly imaged by tilting the sample surface normal from 0° (out-of-plane) to 30° (in-plane) with respect to the beam^36^. The incidence angle in XPEEM is fixed to 74° with respect to the surface. Combined with the surface sensitivity, small tilt angles of the out-of-plane magnetisation could be measured. Accordingly, MTXM and XPEEM allowed for discriminating between in-plane and out-of-plane magnetisation components, and revealing small tilts of the out-of-plane magnetisation, respectively. Magnetic fields up to 30 kA/m were applied perpendicularly to the sample surface. The magnetic state is afterwards visualised at remanence. The observed magnetic contrast is different from that of planar films due to the curved surface. In particular, the XMCD contrast simulations of vortex and donut state type I and type II [Fig. 2(b)] in a magnetic cap reveal a fading of contrast towards the edge of the cap because of decreasing thickness and increasing projection angle. Note that the spin textures are shown for planar disks, while the XMCD contrast is calculated for caps and experimental illumination conditions [see Supplementary for further details on XMCD contrast calculations]. In case of normal incidence and out-of-plane sensitivity, the central region behaves similar to its planar counterpart. An oblique illumination as required for in-plane sensitivity generates in case of an in-plane circulation of the magnetisation (vortex) a dipolar XMCD contrast that is shifted off the center due to the hemispherical geometry of the cap. To avoid complications with contrast analysis of the experimental data, the “upper” part of the projected circle with weak/absent contrast will be shaded.

We focus on samples with a Pd spacer thickness in the range from 1 to 5 nm that show a transition from in-plane to out-of-plane magnetic anisotropy of the Co/Pd spins in XMCD hysteresis loops [Fig. 2(a), also Supplementary Fig. 5]. For *d* = 1 nm, the Py spin configuration is fully imprinted into the Co/Pd layer [Supplementary Fig. 5]. Comparing the experimental with the numerical hysteresis loop suggests that samples with a spacer thickness of 3 ~ 5 nm should have an interlayer coupling strength in the range from 0.4 to 0.5 mJ/m^2^, which stabilises magnetic spiral and donut textures in Co/Pd.

The in-plane magnetisation component of closely packed caps with a 1 nm-thick Pd spacer (indicated by circles) is shown in Figs. 2(c) [also Supplementary Fig. 6] measured at Fe L3 edge (for Py) and Co L3 edge (for Co/Pd); No magnetic out-of-plane contrast were observed. The dipolar XMCD contrast of the Py cap originates from the circulation of the in-plane magnetisation representing the magnetic vortex state as shown schematically in Fig. 2(b). As the sample is tilted by 30° with respect to the photon propagation direction, the magnetic contrast in MTXM appears only in one half of the cap. The other part not revealing physical information is shaded. The vortex core could not be resolved due to the small thickness of Py and the spatial resolution limit. Typically, Py films with a thickness larger than 70 nm are needed to resolve the vortex core^38^,^39^. The magnetic signal in MTXM of the Co layer with a total thickness of only 2 nm is much less than that originating from the 40 nm-thick Py. In this respect, the contrast is enhanced for better visualisation, which also increased the noise. However, the aforementioned dipolar XMCD contrast of a vortex structure in Fe-containing layers is also apparent in the Co signal exhibiting the same circulation [compare Figs. 2(c1) and 2(c2)], as predicted by theory [Fig. 1(c)].

In order to strengthen the statement of an imprinted vortex in the Co/Pd system, samples with Co/Pd layers on top were prepared and visualised by XPEEM, which provides large XMCD signal because of its surface sensitivity [Fig. 2(c3)]. The thin Co/Pd layer and the 1 nm-thick Pd spacer still allow for detecting photoelectrons excited in both Co/Pd and buried Py and thus for direct correlation. Comparing the dipolar XMCD contrast of Co [Fig. 2(c3)] and Fe [Supplementary Fig. 6(b)] reveals planar vortices with same circulation in both subsystems. The vortex state in the thin Co/Pd caps generates a dipolar XMCD contrast contrary to the quadrupolar one occurring in thick Py caps. This is because of the asymmetry in the absorption of left and right circularly polarised X-ray beams while penetrating the magnetic cap is insufficient to compensate the XMCD contrast at the back side of the cap usually inducing an inverted shadow contrast^32^,^33^,^40^. The XMCD contrast in-between neighbouring caps is caused by two mechanisms: Curvature and slight electrostatic charging generate a halo and a detectable magnetic signal even outside the cap; The aforementioned asymmetry in transmitted intensity of left and right circularly polarised light at resonance results in an artificial XMCD contrast of non-magnetic regions (shadow contrast). While the first one sets the same contrast as in the embedding cap, the second one may also partially compensate the XMCD contrast. However, the theoretical prediction of imprinted vortices with same circulation was verified in two different sample systems by two distinct measurement techniques. Note that the comparison between experimental data and simulations performed at *T* = 0 K is valid due to the high Curie temperatures of the layer stack (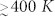).

In case of a 3 nm-thick spacer, both in-plane and out-of-plane magnetisation components were detected in the Co/Pd film [Supplementary Fig. 7]. The corresponding spin texture circulates according to the Py vortex but with a non-vanishing out-of-plane component illustrating a continuous transition between in-plane and out-of-plane Co/Pd magnetisation.

A proper XMCD contrast interpretation becomes even more crucial when analysing more complex spin textures as occurring in samples with thicker Pd spacer, namely 5 nm. The vortex state within the Py subsystem remains stable independently from the interlayer exchange coupling/spacer thickness, as proven by magnetometry measurements [Fig. 2(a)] and XMCD imaging at the Fe edge [Supplementary Fig. 8(a)]. In contrast, Co/Pd spins show a transition towards out-of-plane orientation with increasing spacer thickness [Supplementary Fig. 9]. The residual coherent in-plane magnetisation component due to the imprint mechanism is not detectable by MTXM [Supplementary Fig. 8(b)]. However, its impact can be seen by comparing the XMCD contrast of samples with negligible interlayer coupling (30 nm-thick Pd spacer, Supplementary Fig. 9) and with moderate strength [4 ~ 5 nm, Fig. 2d], revealing “uniform” (out-of-plane saturated magnetisation) and inhomogeneous (unsaturated magnetisation), respectively. The word “uniform” refers to the fact that the XMCD contrast in cap structures fades out when approaching the edge. Figure 2(d1) shows the out-of-plane magnetisation component of the remanent states after initial saturation in a negative out-of-plane magnetic field followed by a small positive one (5 kA/m) visualised by MTXM. Dark and bright contrast refer to out-of-plane magnetisation components aligned parallel and antiparallel to the positive magnetic field direction, respectively. The majority of caps reveal an isotropic contrast with a bright-dark-bright sequence (indicated by cyan circle) representing donut state type II where the inner part of the Co/Pd spins, except for the vortex core, is switched. The occurrence of a few caps with dark-bright contrast (donut I, indicated by red circles) is assigned to an opposite vortex polarity (not switched in saturation field) that leads to an expansion of the core in positive magnetic fields. Please note that deviations from perfect isotropic symmetry of the contrast are due to enhanced contrast and noise.

To provide images with larger signal-to-noise ratio, samples with inverted stack order were investigated by XPEEM. The magnetic states shown in Fig. 2(d2) were recorded at remanence after initially saturating in a negative magnetic field outside the chamber and applying a large positive out-of-plane magnetic field (32 kA/m) that switched all Co/Pd spins but those of the vortex core. The corresponding donut state type I (indicated by a red circle) exhibits a white central region surrounded by a red area. The absence of a blue vortex core is due to the limited spatial resolution of the microscope, which leads to an XMCD signal that refer to a net magnetisation in a certain volume. Thus, the usual implication of white contrast referring to no or very small parallel magnetisation components with respect to the X-ray beam propagation direction as applying to large homogeneous domains with respect to the spatial resolution is not valid. The XMCD contrast of smaller spin textures, such as vortex cores, is blurred and may thus appear white, bluish, blue or even slightly reddish depending on the core size, when surrounded by magnetisation vectors with opposite direction [Supplementary Fig. 10]. In other words, not only the local, but also its spatial distribution as known from shadow contrast analysis is crucial for a proper interpretation. Alternatively, the appearance of states with different central contrast at same field history reflects a distribution of slightly varying magnetic properties throughout the cap array. The small asymmetry in the XPEEM data apparent in each cap and perpendicularly to the beam propagation direction hints for a small in-plane magnetisation component of the donut state due to imprinting. The contrast in-between the caps is due to the same reasons given for Fig. 2(c3). The line profiles of the observed donut states indicate a laterally extended core in the range from 60 to 110 nm [Fig. 2(e)], reliably measured using MTXM and XPEEM with a spatial resolution of 25 nm. These values coincide with those derived from micromagnetic simulations for the expected interlayer exchange coupling, which show a linear increase with decreasing coupling strength [Supplementary Fig. 10]. It has to be noted that donut state type I exhibit a core larger than the vortex core imprinted in Co/Pd by the Py vortex [Fig. 2(d3)]. The remaining states indicated by purple circles in Fig. 2(c2) are swirls/spirals with a slightly tilted out-of-plane magnetisation. The magnetic contrast of the spiral shown in Fig. 2(e) coincides very well with that obtained from simulations. The experimental observation of these states is another proof for interlayer exchange coupling with certain circulation as the vortex core dynamics act as a driven force for the domain wall twisting.

## Manipulation of skyrmion numbers

**Figure 3:**
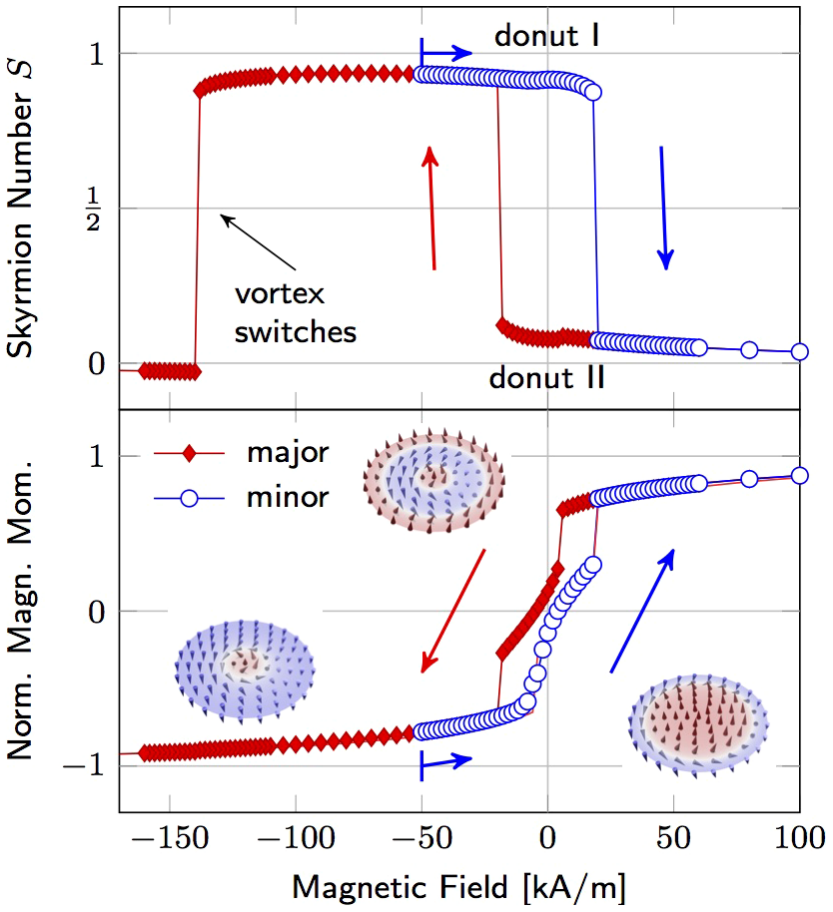
Theoretically predicted switching of skyrmion number *S* *via* out-of-plane magnetic fields (*J_i_* = 0.4 mJ/m^2^). Initially out-of-plane saturated samples (positive field) exhibit donut state type II at remanence with a topological charge close to zero (0.07). At negative fields, all Co/Pd spins except the vortex core reverse, increasing the charge to one until the core switches as well. Setting the field to zero before the core switches results in donut state type I with a nonzero topological charge (0.93). Insets depict the *z* magnetisation component.

The experimental evidence by direct observation of the numerically predicted states, including vortex, donut state type I and II, allows for considering transitions between them. Applying out-of-plane magnetic fields to the sample displaces the magnetic spiral or enlarges the core of the donut state that eventually switches [Fig. 2(d3), also Supplementary Fig. 11]. In this respect, a reliable transformation from donut state type I into type II, and vice versa is achieved with magnetic fields below the switching field of the vortex core. Before mounting, the vortex polarity is set by saturating the sample in a negative out-of-plane magnetic field outside the XPEEM; a large positive magnetic field (32 kA/m) is applied to reverse all Co/Pd spins but those of the vortex core; the field were switched off and the remanent donut state type I were obtained [Fig. 2(d3), left]. Applying a large negative out-of-plane magnetic field (−32 kA/m) followed by a small positive one (5 kA/m) switches the inner Co/Pd spins except for the vortex core revealing donut state type II at the very same magnetic cap [Fig. 2(d3), right]. These transitions were reproduced by micromagnetic simulations using a moderate interlayer exchange coupling (
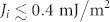
). The larger switching field of the vortex core (140 kA/m) compared to the switching field of Co/Pd (~20 kA/m) allows for setting donut state type I with one domain wall [Fig. 1(d)] by driving a minor hysteresis loop. This state possesses a different skyrmion number compared to donut type II [Fig. 1(c)], since the *z* magnetisation undergoes an odd number of reversals from edge to center resulting in *S* close to 1(0.93) at remanence [Fig. 3]. The corresponding topological charge of donut type II is close to zero (0.07). A similar magnetic field-driven bistability of topologically distinct states, namely single domain and vortex state, was observed in soft-magnetic nanopatterned asymmetric disks^41^. The possibility to switch reliably the skyrmion numbers at remanence is the key advantage of our approach compared to previous works^24^. As our concept does not rely on vortex polarity switching, much lower static magnetic fields are needed to manipulate the topological charge of the material. The small deviation of the skyrmion number from 0 and 1 for donut state type II and type I, respectively, is due to the tilted Co/Pd spins at the edge. This limitation can be overcome by extending the lateral size of the Co/Pd layer and keeping the dimensions of the Py disk unchanged. The modification ensures almost out-of-plane Co/Pd magnetisation at the edge and an asymptotic approach of the net topological charge to integer values. For instance, the same system with a 40 nm-larger Co/Pd diameter has skyrmion numbers of 0.995 and 0.005, but also larger switching fields [Supplementary Fig. 12].

In conclusion, we demonstrated theoretically and verified experimentally the imprint of non-collinear spin textures into an out-of-plane magnetised nanostructure. By continuously varying the interlayer exchange coupling strength, a continuous circulation of the in-plane magnetisation component without Bloch lines was set. This approach allows for engineering skyrmion core textures with adjustable nontrivial skyrmion numbers at room temperature and remanence, which will enable the creation of chiral behaviour/configurations in nanomagnetic systems with a high degree of freedom. The possibility to switch between room temperature stable remanent states with different skyrmion numbers is very promising for digital recording. Although our approach relies on magnetic field-driven manipulation, it can be further extended to current-driven switching as required for future spintronic devices^15^,^16^.

## Methods

### Sample preparation

The patterned layer stacks consist of Pd(2)/[Co(0.4)/Pd(0.7)]_5_/Pd(*d*)/Py(40)/Pd(2) with units in nm and a variable Pd spacer thickness *d* ranging from 1 to 30 nm that were directly prepared onto curvature templates made of assemblies of non-magnetic SiO_2_ spherical particles with a diameter of 500 nm. The multilayer was deposited *via* dc magnetron sputtering at room temperature (base pressure: 7 × 10^−8^ mbar; Ar pressure: 10^−3^ mbar, rate: 1Å/s). By positioning an aperture right above the substrate a curvature-driven thickness gradient is achieved with the nominal thickness at the pole and a persistently decreasing thickness when approaching the equator. To ensure transparency to the soft x-ray radiation for the XMCD spectroscopy and MTXM studies, the samples were fabricated onto 200 nm thin Si_3_N_4_ membranes. Layer stacks with reversed ordering were prepared onto Si wafer and investigated by XPEEM.

### Element-specific characterisation

The magnetic hysteresis loops of the buried Co/Pd multilayer were acquired by means of X-ray magnetic circular dichroism (XMCD) measurements in transmission geometry using the ALICE chamber at the PM-3 beamline (BESSY II). The experimental data were correlated to that of Permalloy *via* switching from Co *L*_3_ (782 eV) to Ni *L*_3_ (861 eV) absorption energy. For in-plane magnetisation reversal, the sample was tilted by 50° with respect to beam and magnetic field direction.

The magnetic domain patterns were imaged using two complementary X-ray microscopy techniques, namely bulk sensitive soft x-ray transmission microscopy (MTXM) at the Advanced Light Source (beamline 6.1.2) in Berkeley, CA, and surface sensitive X-ray magnetic circular dichroism photoemission electron microscopy (XPEEM) at the UE49-PGM1 beamline (BESSY II), providing both a 25 nm spatial resolution. The magnetic contrast is provided by XMCD that detects the difference in X-ray absorption depending on the relative orientation between photon beam and sample surface^36^. In the MXTM, both out-of-plane and in-plane components of the magnetisation can be distinctly imaged by tilting the sample surface normal from 0° (out-of-plane) to 30° (in-plane) with respect to the beam^36^. The incidence angle in XPEEM is fixed to 74° with respect to the surface. Combined with the surface sensitivity, small tilt angles of the out-of-plane magnetisation could be measured. Magnetic fields up to 30 kA/m were applied perpendicularly to the sample surface. The magnetic state were afterwards visualised at remanence.

### Micromagnetic simulations

The imprint of magnetic spin textures was numerically investigated for a disk-like heterostructure consisting of a hard-magnetic out-of-plane magnetised Co/Pd multilayer stack (*M_s_* = 500 kA/m, *A* = 10^−11^ J/m, *K* = 200 kJ/m^3^) and a soft-magnetic Permalloy (*M_s_* = 860 kA/m, *A* = 1.3 × 10^−11^ J/m), which are separated by a non-magnetic spacer of varying thickness *d* [Fig. 1(a)]. The simulations were conducted at *T* = 0 K using Nmag v0.2^42^, a finite-element method/boundary-element method micromagnetic simulator, in combination with the HLib library^43^,^44^. The modeled disk has a diameter of 400 nm with a mesh size of 2.5 nm and 6 nm for Co/Pd (5 nm thick) and Py (40 nm thick), respectively. The effect of the spacer thickness on the coupling between the layers is considered by varying the interlayer exchange coupling strength in the range from 0.1 to 2 mJ/m^2^, as expected for the RKKY-like coupling through Pd^29^,^30^. Landau–Lifschitz-Gilbert damping coefficients of *g_Py_* = 0.05 and *g_Co_* = 0.1 were used for relaxation simulations. The topological charge 
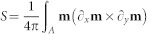
 d*x*d*y* of the Co/Pd spins **m** = *M*/*M_S_* taken over the interface *A* was afterwards calculated from the simulated magnetisation vector field.

## Author Contributions

R.S. and D.M. conceived the experiment. R.S. and G.L. designed and produced the sample. R.S. and D.M. performed the experiments together with M.-Y.I., P.F., F.K., F.R. and R.A. R.S. and L.H. performed micromagnetic simulations. R.S., D.M., U.K.R. and O.G.S. wrote the manuscript considering suggestions from all authors.

## Supplementary Material

Supplementary Information Supplementary Information to: Manipulating Topological States by Imprinting Non-Collinear Spin Textures
