# Supplementary material for: Manipulating Topological States by Imprinting Non-Collinear Spin Textures
Source: Sci Rep. 2015 Mar 5;5:8787. doi: 10.1038/srep08787 (PMC4350102; doi:10.1038/srep08787)
Supplement: Supplementary Information — to: Manipulating Topological States by Imprinting Non-Collinear Spin Textures [file srep08787-s1.pdf]

Supplementary Information to:
Manipulating Topological States by Imprinting Non-Collinear Spin Textures

Robert Streubel[a],[1] Luyang Han,[1] Mi-Young Im,[2,3] Florian Kronast,[4]
Ulrich K. Rößler,[5] Florin Radu,[4] Radu Abrudan,[4,6] Gungun Lin,[1]
Oliver G. Schmidt,[1,7] Peter Fischer[b],[2,8] and Denys Makarov[1]

[1] *Institute for Integrative Nanosciences, IFW Dresden, 01069 Dresden, Germany*

[2] *Center for X-ray Optics, Lawrence Berkeley National Laboratory, Berkeley CA 94720, USA*

[3] *Daegu Gyeongbuk Institute of Science and Technology, Daegu, Korea*

[4] *Helmholtz-Zentrum Berlin für Materialien und Energie GmbH, 12489 Berlin, Germany*

[5] *Institute for Theoretical Solid State Physics, IFW Dresden, 01069 Dresden, Germany*

[6] *Institut für Experimentalphysik/Festkörperphysik,
Ruhr-Universität Bochum, 44780 Bochum, Germany*

[7] *Material Systems for Nanoelectronics, Chemnitz University of Technology, 09107 Chemnitz, Germany*

[8] *Physics Department, UC Santa Cruz, Santa Cruz CA 95064, USA*

The supplementary information supports and extends the conclusions drawn in the manuscript including the following aspects.

(a) The spin configuration of magnetic vortices vertically coupled to out-of-plane magnetised films without interlayer exchange coupling is presented.

(b) The remanent states and the corresponding opening angle of the magnetisation are determined; The core size of the donut configuration is compared to the experimentally determined value.

(c) Magnetic hysteresis loops of individual Co/Pd and Py nanostructures, and of coupled systems with varying interlayer coupling strength are plotted.

(d) The magnetic domain patterns of further samples not shown in the manuscript are listed. Magnetic spin configuration after applying out-of-plane magnetic fields are visualised at remanence by XPEEM. Details on how to calculate the XMCD transmission contrast observed experimentally are given.

[a] r.streubel@ifw-dresden.de
[b] pjfischer@lbl.gov

LIST OF FIGURES

1 Interlayer coupling without interlayer exchange coupling 2

2 Remanent states within Co/Pd multilayers vertically coupled to a magnetic vortex 3

3 Spiral formation in Co/Pd multilayers with an interlayer coupling strength of $0.4\,\mathrm{mJ/m^2}$ 4

4 Experimental magnetic hysteresis loops of Co/Pd and Py cap structures 4

5 Transformation of Co/Pd anisotropy from out-of-plane to in-plane preference revealed by XMCD hysteresis loops 5

6 XMCD contrast of Py and Co/Pd spins with 1 nm Pd spacer visualised by XPEEM 6

7 XMCD contrast of Py and Co/Pd spins with 3 nm Pd spacer visualised by MTXM 6

8 XMCD contrast of Py and Co/Pd spins with 5 nm Pd spacer visualised by MTXM 7

9 XMCD contrast of Co/Pd spins with 30 nm Pd spacer visualised by XPEEM 7

10 Enlarged images of donut states 8

11 Switching behavior of donut and spirals in out-of-plane magnetic field 9

12 Laterally expanded Co/Pd disk approaches integer topological charge. 10

13 Transforming donut state type II into magnetic spiral via in-plane magnetic field 11

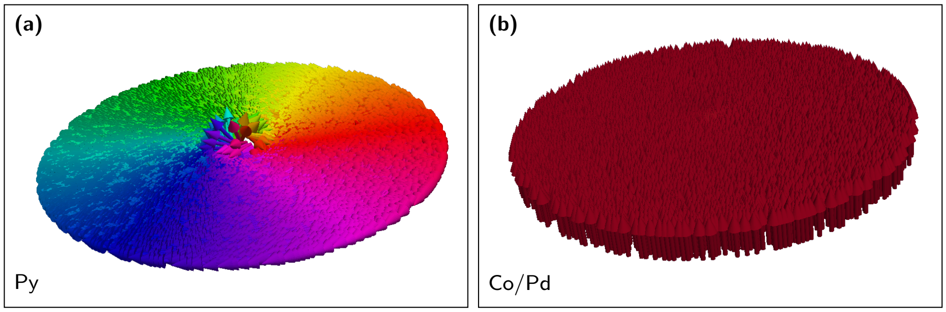

FIG. 1. Micromagnetic calculations on vertically stacked Py and Co/Pd nanostructures with magnetostatic coupling only and without interlayer exchange coupling reveal two decoupled subsystems. (a) Permalloy magnetisation assembles to a planar vortex (color refers to in-plane magnetisation orientation); (b) Co/Pd spins align perpendicularly to the surface.

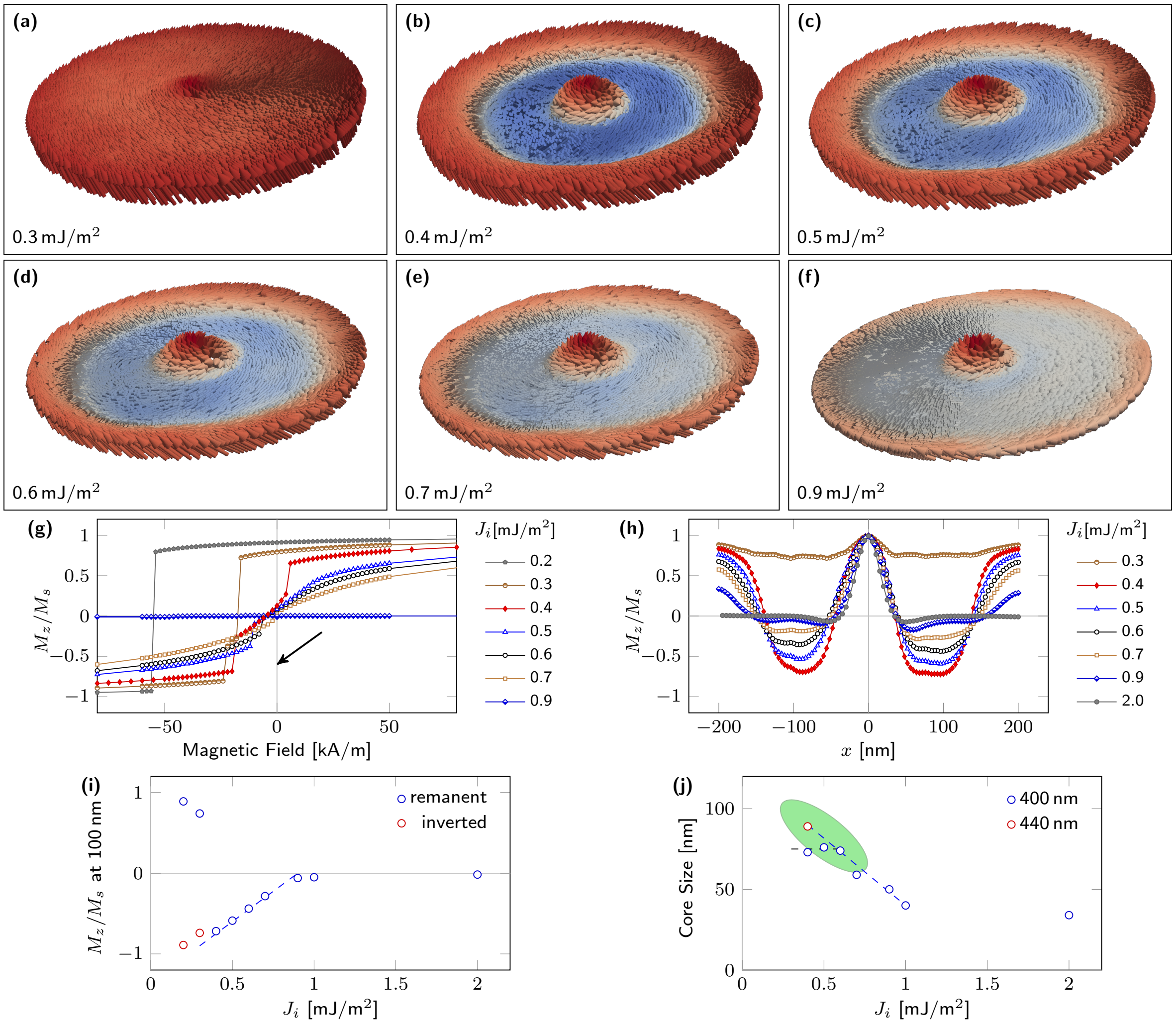

FIG. 2. Remanent states within Co/Pd multilayers (vertically coupled to a magnetic vortex) after applying the magnetic field perpendicular to the surface. Figures (a) – (f) show the magnetisation with colors corresponding to the normalised out-of-plane magnetisation. Initial saturation pointed up (red). For the sake of stray field minimisation, a donut state nucleates with magnetisation pointing down. (g) A certain coupling ($J_i \gtrsim 0.4\,\mathrm{mJ/m^2}$) is required to nucleate a donut state type II (multi-domain state) at remanence. For better visibility, only the hysteresis curve for one direction (from positive fields) is given. In strongly coupled systems, the Co/Pd spins align in the plane following the circulating Py spins. The edge magnetisation reverses at small negative field, while the vortex core is very stable and does not change its polarity in fields smaller than 140 kA/m. (h) Line profile of the out-of-plane magnetisation through the center of the disk. The opening angle of the donut depends on the interlayer exchange coupling. (i) The out-of-plane magnetisation component is linear in the interlayer coupling for intermediate to weak strength. (Red data is multiplied by -1 to illustrate linear trend.) (j) The core size increases linearly for decreasing coupling strength starting at $1\,\mathrm{mJ/m^2}$. For a Co/Pd disk with a diameter of 400 nm, the core size converges ~ 75 nm. A larger diameter (440 nm) allows for a continuation of the linear trend up to 90 nm. The green area indicates the experimentally determined core size for a Pd spacer of 5 nm and a diameter of 500 nm.

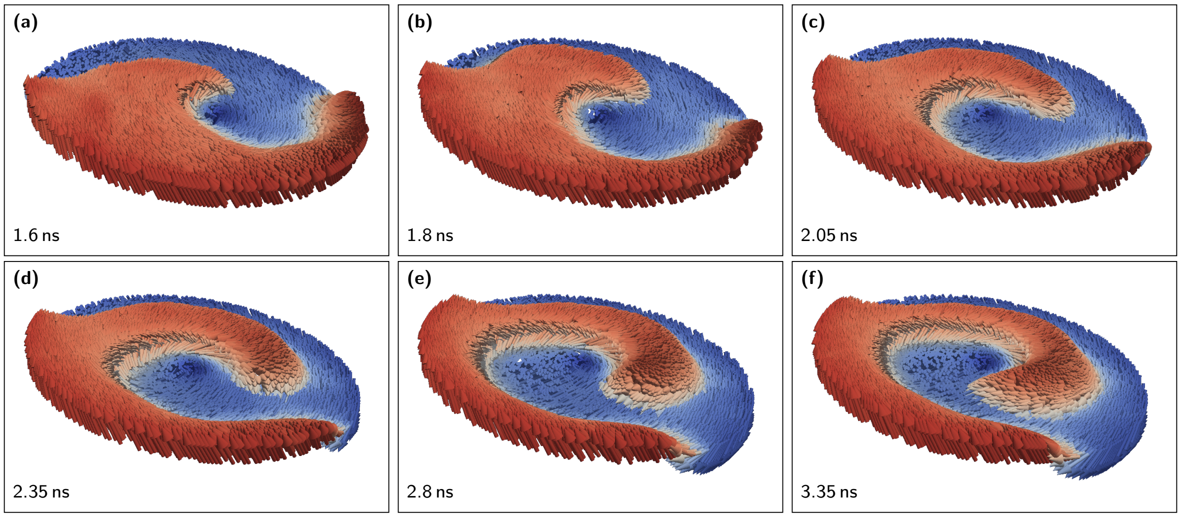

FIG. 3. Spiraling distortion during relaxation of Co/Pd multilayers with an interlayer coupling strength of $0.4\,\mathrm{mJ/m^2}$ after out-of-plane saturation. Figures (a) – (f) show the magnetisation at different time with colors corresponding to the normalised out-of-plane magnetisation. During the relaxation, the system has enough energy to induce a chaotic arrangement that transforms into a two-domain state with a magnetic domain wall reaching from one edge to the other. The intermediate interlayer coupling strength reduces the out-of-plane anisotropy of the Co/Pd system in such a way that a magnetic spiral can be formed. The circulation/twisting coincides with the circulation of the vortex.

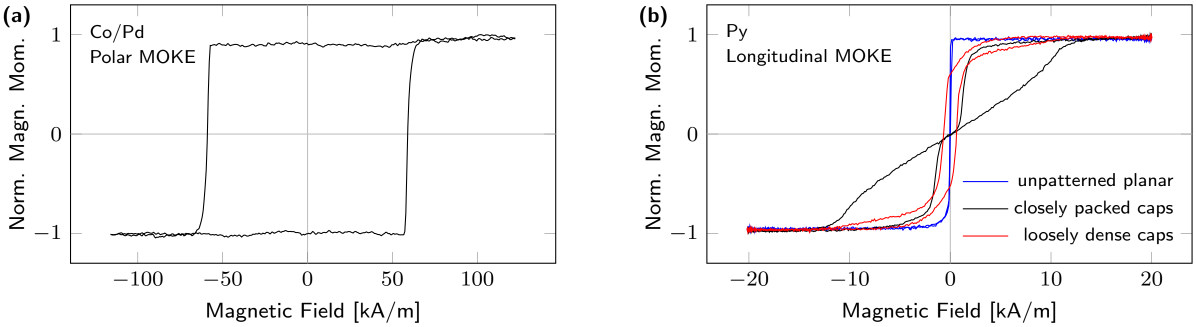

FIG. 4. Magnetic hysteresis loops measured by magnetooptical Kerr magnetometry of (a) $[\mathrm{Co}(0.4)/\mathrm{Pd}(0.7)]_5$ multilayers (out-of-plane) on planar substrate and (b) Permalloy films (40 nm thick) on planar substrate, closely packed cap monolayers ($\varnothing = 500\,\mathrm{nm}$) and loosely dense arrays of caps surrounded by planar film.

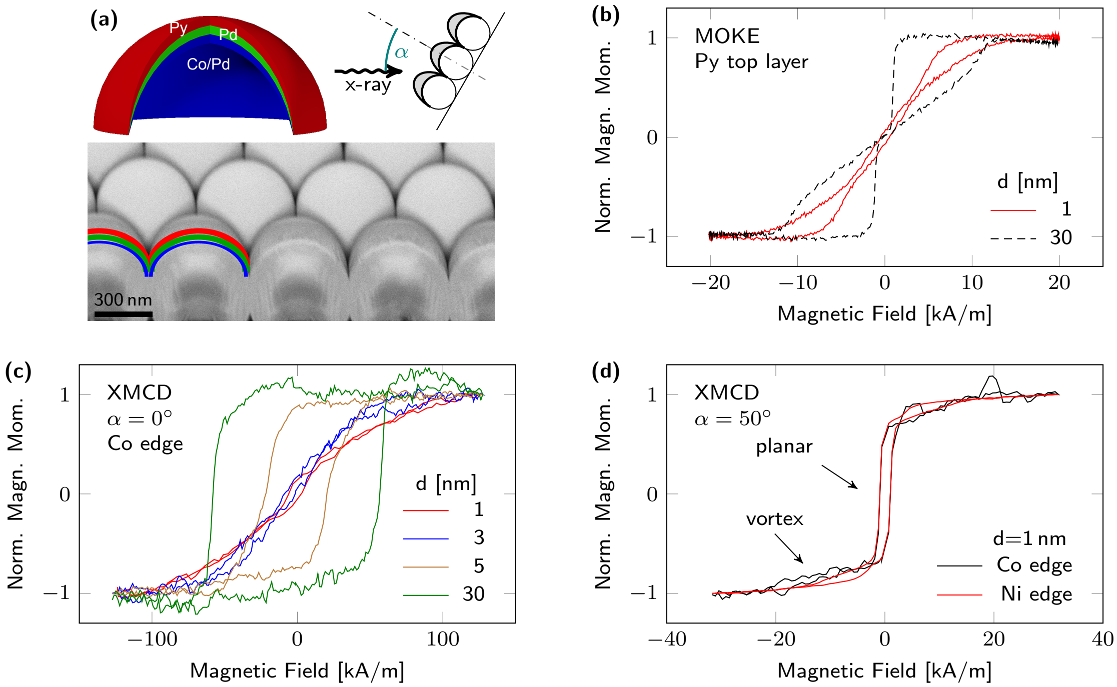

FIG. 5. Realisation of spin texture imprint. (a) Schematics and cross-section SEM of the investigated [Co/Pd]/Pd(d)/Py multilayer stack. (b) Magnetic hysteresis loops of [Co/Pd]/Pd/Py caps obtained by Kerr magnetometry reveal magnetisation in Py top layer and an enhanced vortex stability (smaller vortex nucleation field) for small spacer thickness. (c), (d) Element-specific XMCD hysteresis curves at Co L_3 (Ni L_3) edge of [Co/Pd]/Pd/Py multilayer stacks with different Pd spacer thickness for (c) polar and (d) partially longitudinal (50° tilt) sensitivity. With decreasing spacer thickness the out-of-plane anisotropy of the Co/Pd multilayers weakens. The interlayer exchange coupling favors an in-plane alignment of the Co/Pd spins. The measured hysteresis is dominated by the magnetisation of the planar film, as the sparsely distributed small caps contribute little.

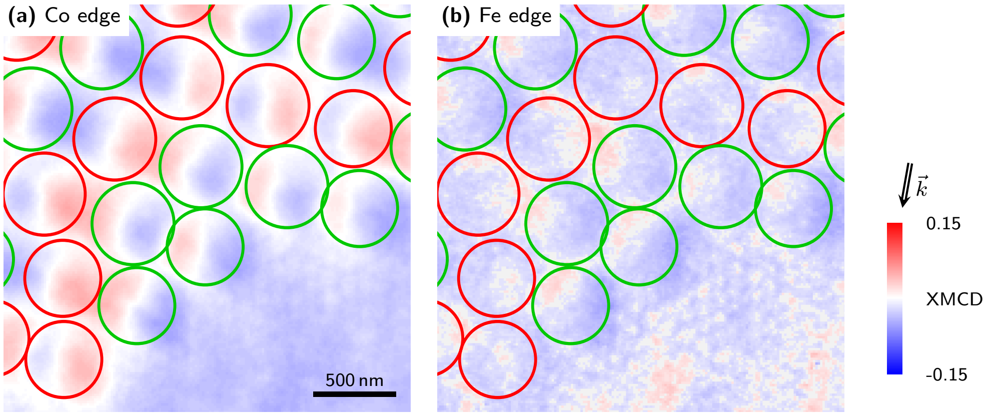

FIG. 6. Magnetic domain patterns of Co/Pd spins coupled to Py vortices and separated by a 1 nm-thick Pd spacer visualised by XPEEM. The strong interlayer exchange coupling leads to a parallel alignment of the Co/Pd spins with respect to the Py spins. As the Py cap is buried underneath the Co/Pd system, a weak but obvious XMCD contrast is recorded that is confined to the front side of the cap. The circulation of the in-plane magnetisation is indicated by green and red circles.

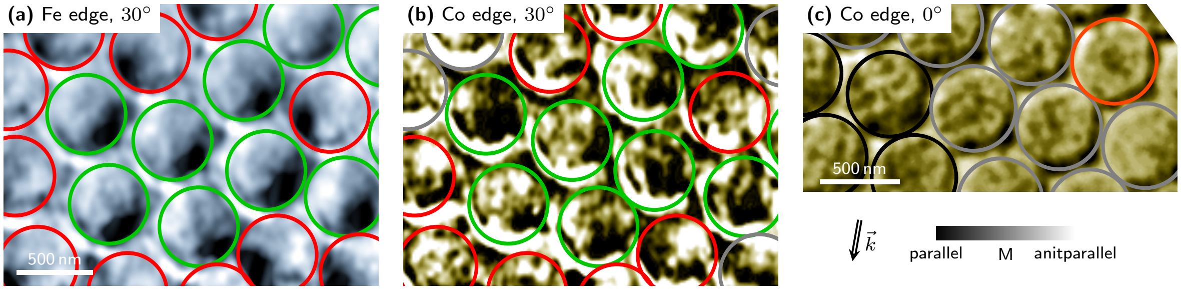

FIG. 7. Magnetic domain patterns of Co/Pd spins coupled to Py vortices and separated by a 3 nm-thick Pd spacer visualised by MTXM. The intermediate interlayer coupling leads to a significantly tilted magnetisation with both in-plane and out-of-plane components. Panels (b) and (c) are take at different locations. The circulation of the in-plane magnetisation is indicated by green and red circles.

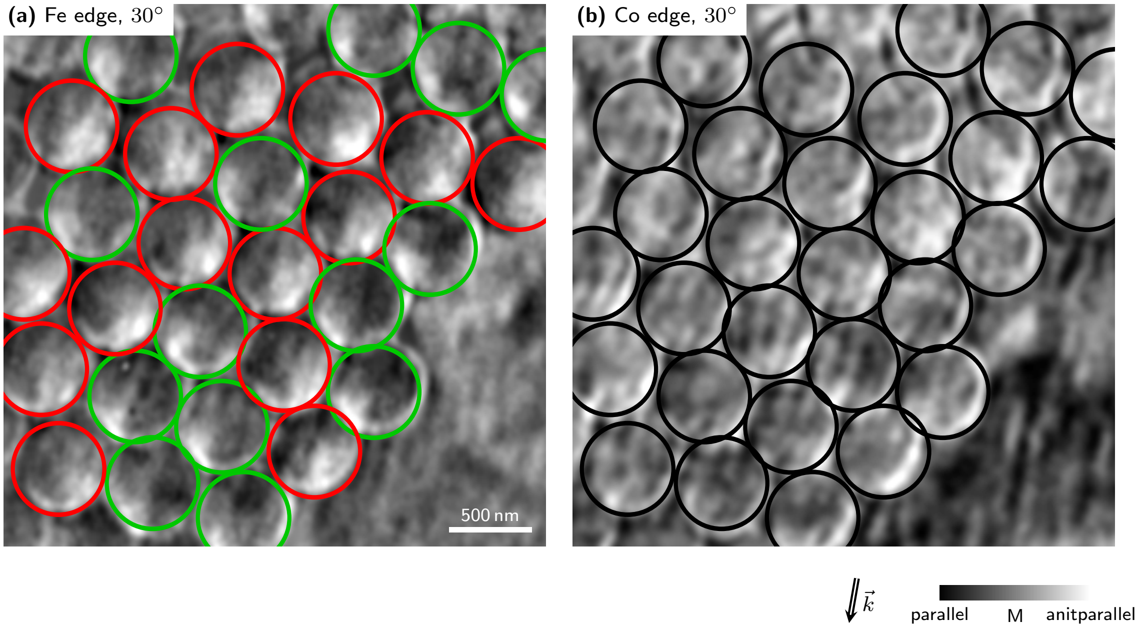

FIG. 8. (a) The vortex state of the Py system does not change within the experimental uncertainty of MXTM by varying the Pd spacer thickness (here shown for 5 nm spacer). (b) As the tilt of the Co/Pd spins with respect to the Py spins increases with increasing spacer thickness, no detectable XMCD signal could be observed. The circulation of the in-plane magnetisation is indicated by green and red circles.

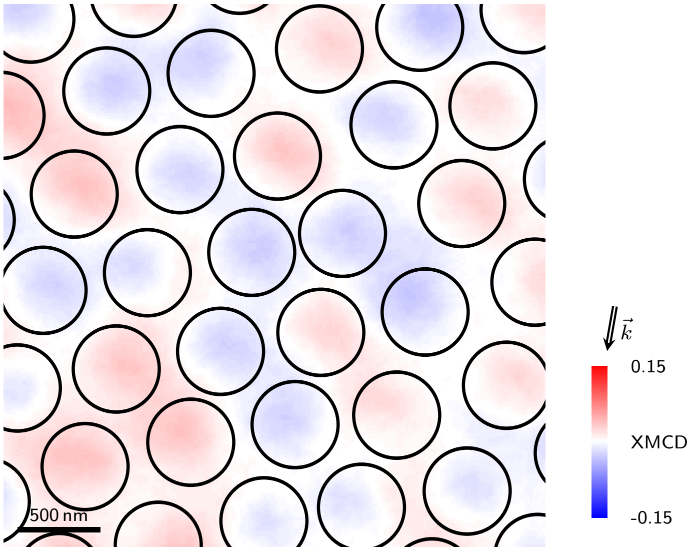

FIG. 9. A large separation of 30 nm between the Co/Pd spins and the Py vortices does not alter the originally out-of-plane saturated state of the Co/Pd system due to negligible interlayer exchange coupling. Nor does it allows for forming donut states for the given geometry.

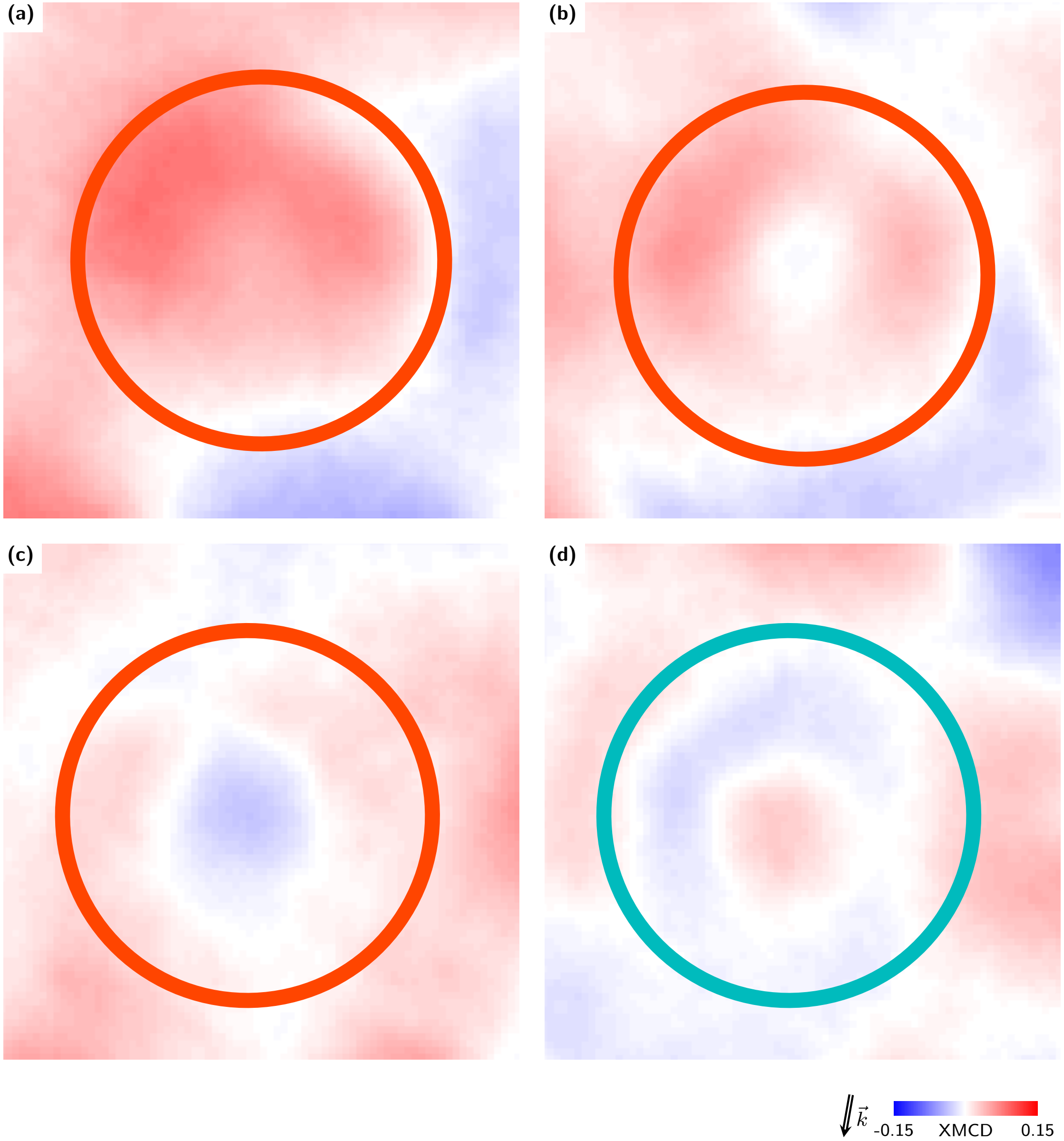

FIG. 10. Enlarged XMCD images of donut states in Co/Pd visualised at remanence in XPEEM. The sample was originally saturated in a negative out-of-plane magnetic field (blue contrast). By applying positive magnetic fields of various field strength, all Co/Pd spins but those of the vortex core switch (red contrast). Depending on the core size (and magnetic field), the contrast of the core is (a) slightly white, (b) blueish or (c) dark blue. A shift of the central region to the blue scale occurs in any case. (d) Donut state type II shows a light red contrast that is surrounded by the red and blue circles.

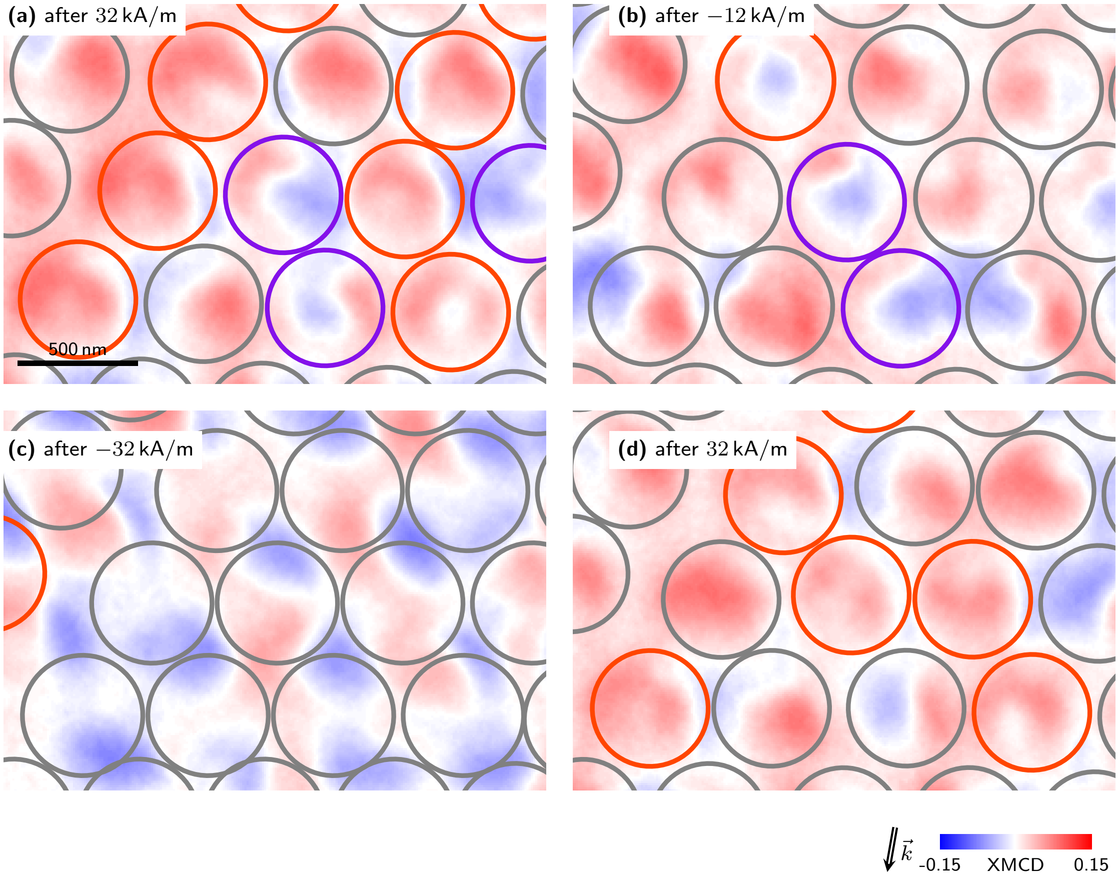

FIG. 11. Remanent states of Co/Pd spins separated by 4 nm Pd from the Py vortex after applying various out-of-plane magnetic fields visualised by XPEEM. Red and purple circles indicate donut and spiral state, respectively. As the field becomes larger, the donut state transforms by extending the inner domain to the edge. A slight asymmetry between positive and negative fields is apparent, which is assigned to the influence of the unswitchable vortex core (blue contrast).

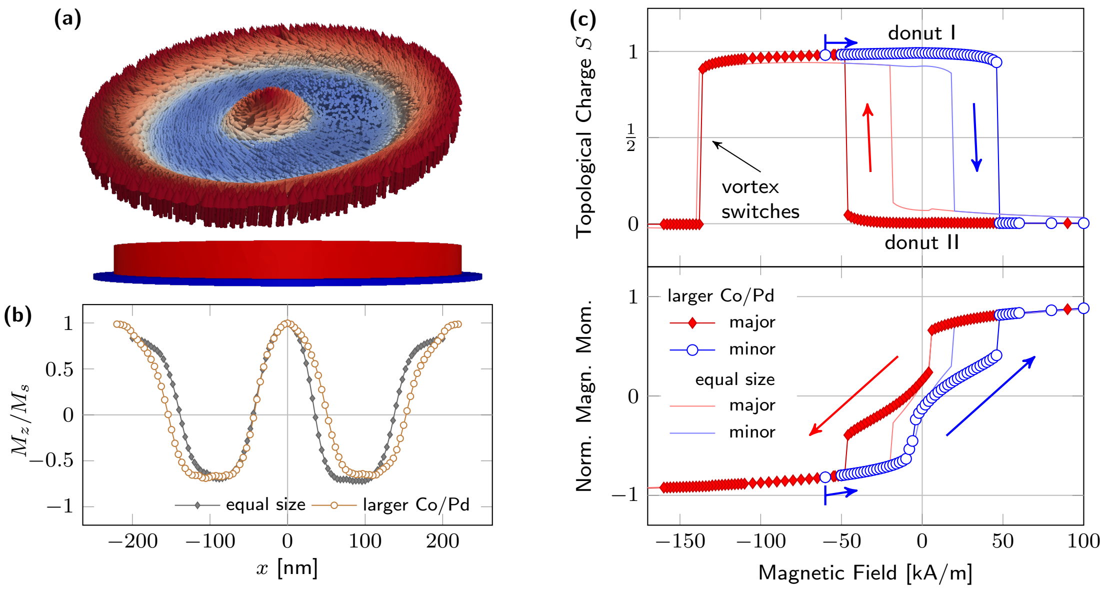

FIG. 12. Lateral expansion of the Co/Pd disk by less than 20 nm ensures out-of-plane magnetisation at the edge and thus an integer topological charge. (a) Remanent state after driving an out-of-plane magnetic field. Schematic illustrate the geometry used for calculation: Py disk (red) and Co/Pd disk (blue). (b) Line profiles of the out-of-plane magnetisation through the center of the disk with same interlayer exchange coupling ($J_i = 0.4\,\mathrm{mJ/m^2}$). (c) The topological charge increases (decreases) from 0.93 (0.07) to 0.995 (0.005) by laterally expanding the Co/Pd disk from 400 to 440 nm in diameter. The switching fields increase as well.

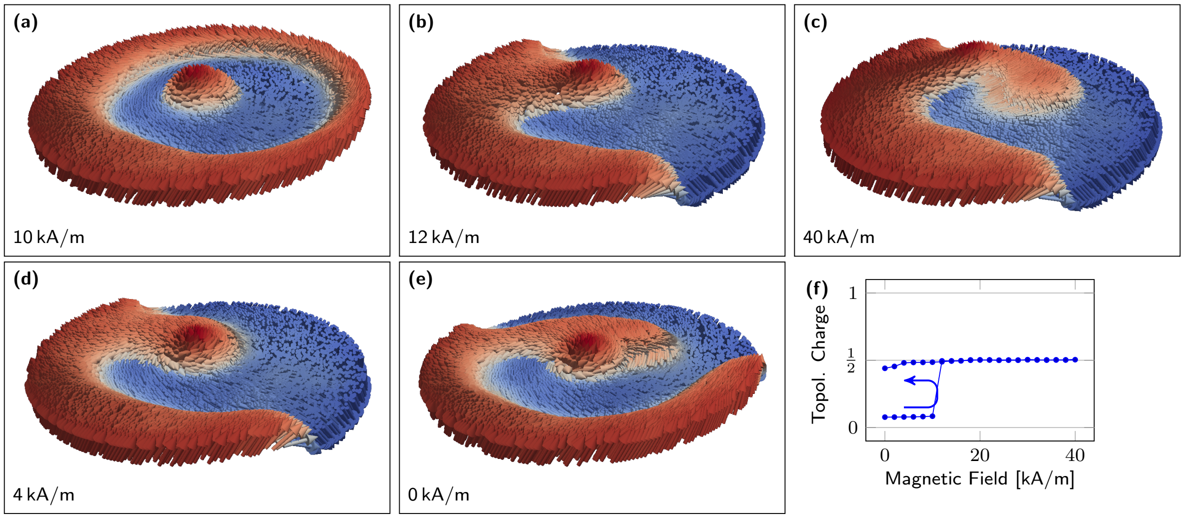

FIG. 13. Transforming donut state type II into magnetic spiral via in-plane magnetic field ($J_{\mathrm{inter}} = 0.4\,\mathrm{mJ/m^2}$). Figures (a) – (e) depict snapshots of the hysteresis loop with colors corresponding to the normalised out-of-plane magnetisation. The vortex core is displaced according to the in-plane magnetic field. (b) At 11 kA/m, the domain wall of the donut state breaks and the magnetic spiral emerges with its tip coinciding with the vortex core. (c) As the core approaches the edge, the spiral jumps back to the center to reduce the stray field. (e) In the remanent state, the vortex core is again at the tip of the spiral. (f) The transition also changes the topological charge, which switches from zero to $\frac{1}{2}$.

Calculation of XMCD contrast of a magnetic cap

The magnetic cap is approximated as the upper part of the volume enclosed by a sphere with a diameter of 500 nm and an ellipsoid with 500 and 510 nm elongations. Its discretisation is $1 \times 4 \times 4\,\mathrm{nm}^3$. The magnetic states in a cap are described analytically by projecting the numerically derived state in disk geometry onto a hemisphere. The absorption is calculated with the case of dipole transition and thus depends on the scalar product between magnetisation and X-ray beam.

Topologically stable magnetisation states [1]

In this paper, we use a fractional "topological charges" to classify different magnetisation configuration in finite film elements. In this context, it is noteworthy that these topological charge densities do not strictly define topologically stability of these states owing to the open boundaries of these elements.

For a consistent definition of topological states, the boundary of the physical space *at infinity* must identified with with a single point so that the pre-images of loops or surfaces cannot be deformed away. Mathematically, this construction is called *compactification.* As a pertinent example, a two-dimensional magnetised film is described by first mapping its physical space to a sphere through a stereographic transformation which identifies the coordinate origin with the south-pole and the locations at infinity with the north pole of the spherical surface. In order to construct the homotopy groups and to find non-trivial states, the set of continuous maps have to be considered between loops or closed surfaces on this sphere to another sphere that describes the magnetisation. The maps from the sphere describing the physical space to the sphere describing the magnetisation directions can be employed to find non-trivial topologies.

An integer number counts how often this maps covers the sphere of order-parameter directions for a magnetisation configuration. This construction of a number yields the topological index. In fact, this integer is the skyrmion number of the magnetisation distribution. From this elementary construction, it is seen that an integer topological index for a magnetisation distribution in a circular two-dimensional disk cannot be defined. The calculation of integrals over the "topological charge densities" will yield fractional numbers corresponding to the solid-angle area covered by the magnetisation on the order-parameter sphere. This rational numbers can continuously be changed by deformations of the magnetisation distribution (or of the physical space, i.e. the magnetic disk).

For further information, see, e.g., [2].

[1] Mermin, N. D. The topological theory of defects in ordered media. *Rev. Mod. Phys.* **51**, 591–648 (1979).

[2] Eschrig, H. Topology and Geometry for Physics, in Lecture Notes in Physics. *Springer* **822** (2011).
